# Volumetric modulated arc therapy treatment planning of thoracic vertebral metastases using stereotactic body radiotherapy

**DOI:** 10.1002/acm2.12252

**Published:** 2018-01-19

**Authors:** Matthew Mallory, Damodar Pokhrel, Rajeev Badkul, Hongyu Jiang, Christopher Lominska, Fen Wang

**Affiliations:** ^1^ Department of Radiation Oncology The University of Kansas Cancer Center Kansas City KS USA; ^2^ Department of Radiation Medicine University of Kentucky Chandler Hospital Lexington KY USA

**Keywords:** SBRT, Spine, VMAT

## Abstract

**Purpose/Objectives:**

To retrospectively evaluate the plan quality, treatment efficiency, and accuracy of volumetric modulated arc therapy (VMAT) plans for thoracic spine metastases using stereotactic body radiotherapy (SBRT).

**Materials/Methods:**

Seven patients with thoracic vertebral metastases treated with noncoplanar hybrid arcs (NCHA) (1 to 2 3D‐conformal partial arcs +7 to 9 IMRT beams) were re‐optimized with VMAT plans using three coplanar arcs. Tumors were located between T2 and T7 and PTVs ranged between 24.3 and 240.1 cc (median 48.1 cc). All prescriptions were 30 Gy in 5 fractions with 6 MV beams treated using the Novalis Tx linac equipped with high definition multileaf collimators (HDMLC). MR images were fused with planning CTs for target and OAR contouring. Plans were compared for target coverage using conformality index (CI), homogeneity index (HI), D90, D98, D2, and Dmedian. Normal tissue sparing was evaluated by comparing doses to the spinal cord (Dmax, D0.35, and D1.2 cc), esophagus (Dmax and D5 cc), heart (Dmax, D15 cc), and lung (V5 and V10). Data analysis was performed with a two‐sided t‐test for each set of parameters. Dose delivery efficiency and accuracy of each VMAT plan was assessed via quality assurance (QA) using a MapCHECK device. The Beam‐on time (BOT) was recorded, and a gamma index was used to compare dose agreement between the planned and measured doses.

**Results:**

VMAT plans resulted in improved CI (1.02 vs. 1.36, *P* = 0.05), HI (0.14 vs. 0.27, *P* = 0.01), D98 (28.4 vs. 26.8 Gy, *P* = 0.03), D2 (32.9 vs. 36.0 Gy, *P* = 0.02), and Dmedian (31.4 vs. 33.7 Gy, *P* = 0.01). D90 was improved but not statistically significant (30.4 vs. 31.0 Gy, *P* = 0.38). VMAT plans showed statistically significant improvements in normal tissue sparing: Esophagus D_max_ (22.5 vs. 27.0 Gy, *P* = 0.03), Esophagus 5 cc (17.6 vs. 21.5 Gy, *P* = 0.02), and Heart D_max_ (13.1 vs. 15.8 Gy, *P* = 0.03). Improvements were also observed in spinal cord and lung sparing as well but were not statistically significant. The BOT showed significant reduction for VMAT, 4.7 ± 0.6 min vs. 7.1 ± 1 min for NCHA (not accounting for couch kicks). VMAT plans demonstrated an accurate dose delivery of 95.5 ± 1.0% for clinical gamma passing rate of 3%/3 mm criteria, which was similar to NCHA plans.

**Conclusions:**

VMAT plans have shown improved dose distributions and normal tissue sparing compared to NCHA plans. Significant reductions in treatment time could potentially minimize patient discomfort and intrafraction movement errors. VMAT planning for SBRT is an attractive option for the treatment of metastases to thoracic vertebrae, and further investigation using alternative fractionation schedules is warranted.

## 1 INTRODUCTION

The spinal vertebrae are a common site of metastasis for many cancers and can cause significant pain and neurologic dysfunction.^1^ Each year, nearly 20,000 new cases of spinal metastases are diagnosed in North America with an annual prevalence of around 100,000 cases.^2^, ^3^, ^4^ Bone metastases are common in many different solid tumors with up to 90 percent of patients with breast or prostate cancer having osseous disease in autopsy studies.^5^, ^6^ Pathologic fractures in the spine from metastases are painful and debilitating resulting in poor quality of life, which is an important consideration in the setting of recent life‐prolonging advances in systemic treatment for metastatic disease.^7^, ^8^, ^9^, ^10^ For these reasons, palliative local management will play an ever more crucial role in the treatment of metastatic cancer involving the spine.

Randomized data support the combination of surgery and radiation in cases of spinal cord compression; however, the optimal management for spinal metastases without cord compression is still unclear.^11^ Radiation offers the advantage of sparing the patient from an invasive procedure, but conventional techniques are limited by the tolerance dose of the spinal cord.^12^ Conventional single fraction treatments (BED ~14 Gy_10_) and multifraction treatments (BED ~30–40 Gy_10_) have demonstrated low clinical complete response rates and suboptimal tumor control. Retreatment is often required, especially for single fraction treatments, and may be precluded by prior radiation leaving surgery as the only option.^13^, ^14^, ^15^, ^16^

SBRT has emerged as an attractive method of dose escalation (BED ~40–80 Gy_10_) while respecting spinal cord tolerance through advanced planning techniques using image‐guided (IG) intensity‐modulated radiation therapy (IMRT).^17^, ^18^, ^19^, ^20^, ^21^ SBRT can be delivered on multiple platforms with IMRT including multileaf collimator (MLC) equipped on most treatment units, TomoTherapy, or CyberKnife. Delivery of SBRT to the vertebrae while avoiding the spinal cord typically requires the generation of complex hybrid plans consisting of noncoplanar partial arcs and static IMRT beams. While these methods produce highly conformal plans with a higher BED and shorter overall treatment time as compared to traditional two‐dimensional palliative dose regimens, the individual treatments are lengthy and require a large number of total monitor units.^22^

Methodology to minimize BOT is an area of interest for SBRT to the spine due to concerns over intrafraction motion and patient comfort. Volumetric Modulated Arc Therapy (VMAT) is an elegant technique of delivering IMRT that allows for shorter treatment times achieved by optimizing MLC positions and dose rate while the gantry rotates around the patient with the beam‐on.^23^, ^24^, ^25^ SBRT to the spine has been demonstrated to be feasible and safe in a phase I study; however, concerns over the possibility of vertebral compression fracture and radiation‐induced myelitis remain and are an active area of investigation.^26^, ^27^, ^28^ Techniques for planning and immobilization are of special interest in regards to limiting toxicity by keeping treatment times and intrafraction motion to a minimum.^29^, ^30^ In this report, we retrospectively evaluate VMAT plans for thoracic spinal metastases using SBRT in terms of plan quality, treatment efficiency, and accuracy.

## 2 MATERIALS AND METHODS

### 2.A Hybrid planning and treatment procedure

For this retrospective study, we replanned seven patients in the Eclipse version 11.0 treatment planning system (Varian, Palo Alto, CA, USA). These patients were previously treated at our institution for thoracic vertebral metastases with SBRT using iPlan (BrainLAB, Feldkirchen, Germany). CT simulations were performed on a 16 slice Phillips Brilliance Big Bore CT Scanner. Highly conformal SBRT treatment plans were generated using noncoplanar hybrid arcs (a combination of 3D noncoplanar conformal arcs and nonopposing static beams). Treatments were delivered with a Novalis TX linear accelerator (Varian, Palo Alto, CA, USA) using 6 MV beams (600 MU/min) and a HDMLC. The HDMLC on this machine consisted of 120 leaves (30 pairs of 2.5 mm leaves surrounded by 30 pairs of 5 mm leaves). No additional margin for dose buildup was applied at the edges of the MLC blocks beyond the PTV. All treatment plans were calculated using the pencil‐beam algorithm with heterogeneity corrections turned on with 2.0 × 2.0 × 2.0 mm^3^ dose grid sizes. A Monte Carlo algorithm is also available and is used for lung SBRT treatments in our clinic; however, based on clinical experience there are no significant differences with the Monte Carlo algorithm for spinal SBRT, and the pencil‐beam algorithm is standardly used for these treatments. The treatment prescription was 30 Gy in 5 fractions with at least 90% of the PTV encompassing 100% of the prescription isodose. Immobilization was accomplished with a BodyFIX double‐vacuum immobilization device (Elekta, Stockholm, Sweden) and abdominal compression. The ExacTrac system from BrainLAB was utilized for initial patient setup. Quality assurance checks were performed daily in order to ensure accurate target localization. Prior to each treatment, a pair of oblique kV x‐ray images was acquired and automatic 2D/3D image registration was performed in the ExacTrac system. Cone beam CT scans were then performed with Varian onboard imaging (OBI). All quality assurance procedures were in compliance with the standard SBRT treatment delivery technique following AAPM guidelines.^27^, ^28^ Specifically, the Winston‐Lutz test was performed daily before SBRT treatments confirming coincidence of the radiation isocenter and mechanical isocenter.^31^

### 2.B VMAT planning

After obtaining institutional review board approval from our institution, all DICOM 3D‐CT datasets and contoured structures for the seven treated patients were electronically transferred from BrainLAB iPlan workstation to Eclipse treatment planning system for the purpose of replanning and optimization using the VMAT technique. All VMAT plans were generated for use with a 6 MV beam on a Novalis TX linear accelerator equipped with HDMLCs (2.5 mm leaf width at isocenter) with a maximum dose rate of 600 MU/min. The isocenter was placed at the center of the PTV in the beam's eye view. Three full coplanar arcs covered 358° gantry rotation. The first clockwise arc used a 25° collimator rotation, and the counter clockwise arc used a complementary 335° rotation. The second clockwise arc used 55° collimator rotation to reduce the overlapping MLC tongue‐and‐groove leakage for VMAT plans. All treatment plans were generated in Eclipse TPS using anisotropic analytical algorithm (AAA) for heterogeneity corrections with 2.0 × 2.0 × 2.0 mm^3^ dose grid sizes for dose calculations. Each plan had a dose delivery schema of 30 Gy in 5 fractions with at least 90% of the PTV receiving the prescription dose (D_90%_ ≥30 Gy). Gantry speed, dose rate, and MLC motion were optimized in VMAT plans using the inverse optimization algorithm in the Eclipse TPS to achieve desired dose distributions.

### 2.C Evaluation of dose distribution

Dose volume histograms were generated for all hybrid and VMAT treatment plans in the Eclipse TPS for the PTV, spinal cord, esophagus, and heart. Dosimetric evaluation of these plans was performed by calculating conformality index (CI) and heterogeneity index (HI) using the DVH of the PTV.

The *CI* as defined per ICRU is:

(1) $$CI=\frac{V_{\mathrm{ip}}}{V_{\left(t\arg et\right)}}$$

where *V*_*ip*_ represents the treated volume enclosed by the prescription isodose line and *V*_*(target)*_ represents the target volume for the PTV. CI values near unity indicate superior plan conformity of dose distribution to the target volume.

The *HI* as defined per ICRU is:

(2) $$HI=\frac{\left(D_{2\%}-D_{98\%}\right)}{D_{\mathrm{median}}}$$

where D_2%_ and D_98%_ correspond to the dose delivered to 2% and 98% of the PTV, respectively, and D_*median*_ represents the median dose to the PTV. Smaller values of HI indicate better dose homogeneity within the target volume.

Each hybrid and VMAT plan was evaluated for PTV coverage and dose to the OARs (spinal cord, esophagus, and heart).

### 2.D Efficiency and dose delivery accuracy

**Figure 1:**
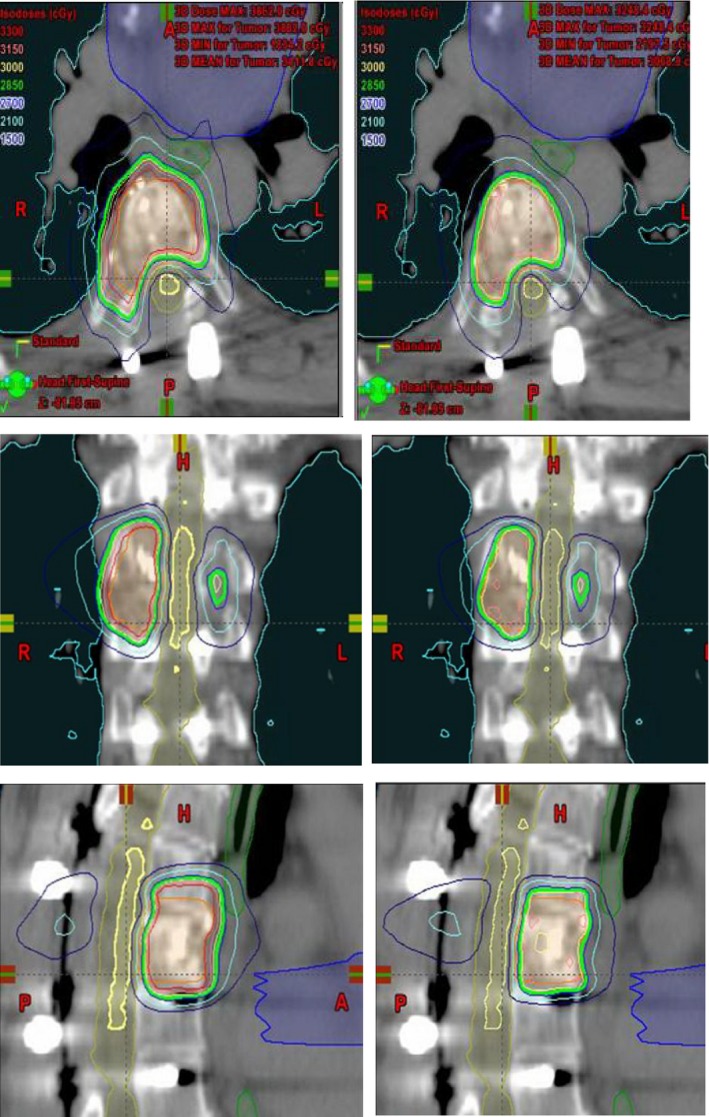
Comparison of isodose distributions and the DVHs for NC‐HA (left) and RapidArc (right) VMAT planning in the same case shown in axial, coronal, and sagittal views. Planning target volume (PTV) is shown in orange and partial‐spinal cord in yellow. Isodose lines are shown as colorwash (30 Gy, orange; 28.5 Gy green; 21 Gy light‐blue and 15 Gy dark‐blue). The other critical structures such as esophagus, heart and lung contours are also shown in the 3D image views. [Correction added on 8^th^ February 2018, after first online publication: Caption was corrected.]

The dose delivery efficiency of each plan was evaluated in terms of total number of MUs and actual BOT, which was recorded at the treatment console while delivering the QA plan. Delivery accuracy of the QA plan was assessed by physically measuring the 2D dose distribution of each plan on an in‐house static plastic phantom which housed the MapCHECK device (Sun Nuclear Corporation.; Melbourne, FL, USA). The plastic phantom was made up of 30 × 30 × 20 cm^3^ in dimensions provided buildup of 10 cm at the top and bottom as well as 5 cm on all other sides. All QA plans were delivered at the machine in one session, minimizing dependence of the QA passing rates on machine output. The measured cumulative 2D dose plane computed by the Eclipse treatment planning system (version 11.0) was compared with the measured dose using the MapCHECK QA device inserted in the middle of plastic phantom as shown in Fig. 1. Upon completion of delivered dose, data were analyzed with MapCHECK software (SNC patient, version 6.1) using the Van Dyk gamma passing rate criteria of 3/3 (3%/3 mm).

## 3 RESULTS

**Figure 2:**
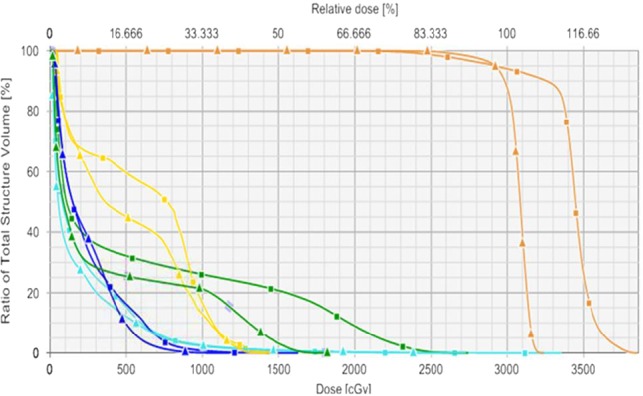
Comparison DVHs for NC‐HA and RapidArc VMAT planning in the same case shown in axial, coronal, and sagittal views. The DVHs NC‐HA (square) and RapidArc VMAT (triangle) clearly shows significant dosimetric advantages when using VMAT planning for thoracic vertebral SBRT. Contours and corresponding DVHs represented: PTV (orange), heart (dark‐blue), esophagus (dark‐green), spinal cord (yellow), and lung (light‐blue). [Correction added on 8^th^ February 2018, after first online publication: Caption was corrected.]

**Figure 3:**
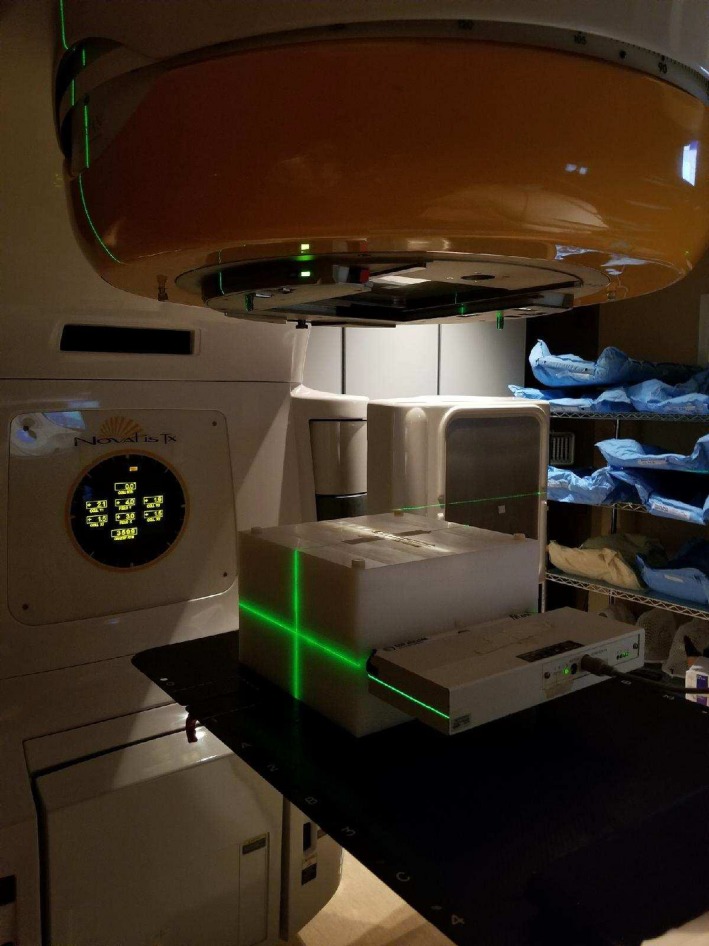
Experimental QA setup in treatment room showing the MapCHECK QA device inserted in the middle of plastic phantom. [Correction added on 8^th^ February 2018, after first online publication: Caption was corrected.]

Computed dose distributions for both hybrid (Hyb) and VMAT (Rapid) plans in coronal, sagittal, and axial views for one representative patient are shown in Fig. 2. The corresponding DVHs for both plans of the same patient are also shown in Fig. 3.

Table 1 presents the dosimetric results of all seven hybrid and VMAT plans. VMAT plans demonstrated excellent conformality reflecting improvement in the conformality index from a mean value of 1.36 ± 0.40 for hybrid plans to 1.02 ± 0.04 for VMAT plans (*P* = 0.05). Dose homogeneity also improved, evidenced by D2 and D98 values closer to prescription dose, which resulted in the tightening of the homogeneity index from 0.27 ± 0.09 for hybrid plans to 0.14 ± 0.05 for VMAT plans (*P* = 0.01). PTVD90 was improved for VMAT plans, although not to a statistically significant level (30.4 ± 0.3 vs. 31.0 ± 0.2 Gy, *P* = 0.38).

**Table 1:** Conformality Index (CI), Homogeneity Index (HI), and PTV coverage in terms of D98, D90, D2, and PTVD90

Patient no	Hybrid plan						VMAT plan					
	D2% (Gy)	D98% (Gy)	D_median_ (Gy)	HI	CI	PTVD90 (%)	D2% (Gy)	D98% (Gy)	D_median_ (Gy)	HI	CI	PTVD90 (%)
I	34.3	29.9	32.8	0.13	1.05	31.1	32.7	30.0	31.1	0.09	1.03	30.5
II	39.1	25.6	35.2	0.38	1.11	30.5	32.5	28.0	31.4	0.14	1.04	30.3
III	35.3	29.0	33.6	0.19	1.13	30.7	35.0	29.1	32.2	0.18	0.98	30.8
IV	38.4	26.8	35.9	0.32	1.07	33.7	32.9	29.5	31.8	0.11	0.99	30.8
V	33.8	24.5	31.5	0.30	1.98	29.9	32.5	28.7	31.2	0.12	1.04	30.3
VI	37.3	26.3	34.5	0.32	1.88	32.6	31.8	27.6	30.9	0.14	1.07	30.3
VII	33.6	25.3	32.4	0.26	1.33	28.5	32.9	25.9	31.6	0.22	0.96	30.1
Mean	36.0 ± 2.3	26.8 ± 2.0	33.7 ± 1.6	0.27 ± 0.09	1.36 ± 0.40	31.0 ± 0.2	32.9 ± 1.0	28.4 ± 1.4	31.5 ± 0.4	0.14 ± 0.05	1.02 ± 0.04	30.4 ± 0.3

Tables 2 and 3 present the dosimetric data for the organs at risk (OARs) for hybrid and VMAT plans, respectively. Maximum doses to the spinal cord, esophagus, and heart are shown along with D0.3 and D1.2 cc for the spinal cord, D5 cc for the esophagus, and D15 cc for the heart. The volume of the lung receiving no more than 5 and 10 Gy is shown along with the minimum dose to the hottest 1000 cc of lung (D1000 cc) as an indicator of low dose spread. For VMAT plans compared to hybrid plans, the average maximum dose to the esophagus improved from 27.0 to 22.5 Gy (*P* = 0.03), and the average maximum dose to heart improved from 15.8 to 13.1 Gy (*P* = 0.03). Similarly, average esophagus dose to 5 cc volume improved from 21.5 to 17.6 Gy (*P* = 0.02). There was a trend for improvement in spinal cord and lung sparing, but these findings were not statistically significant. The average cord max was 19.9 Gy for hybrid plans and 17.8 Gy for VMAT plans (*P* = 0.16). The average lung V5 was 19.2 for hybrid plans and 18.5 Gy for VMAT plans (*P* = 0.42).

**Table 2:** Dose to organs at risk for hybrid plans for all seven patients

Patient no	Hybrid plan									
	Cord max (Gy)	Cord 0.3 cc (Gy)	Cord 1.2 cc (Gy)	Esophagus max (Gy)	Esophagus 5 cc (Gy)	Heart max (Gy)	Heart 15 cc (Gy)	Lung V5 (cc)	Lung V10 (cc)	Lung D1000 cc (cGy)
I	11.9	10.4	6.5	13.3	8.6	14.1	7.9	10.1	2.9	78
II	21.2	20.3	19.3	30.7	25.3	4.4	5.4	6.7	2.7	22
III	34.3	23.2	22.4	32.8	29.3	21.4	10.9	53.1	18.6	735
IV	15.0	12.8	12.0	20.2	15.8	12.7	3.5	7.8	2.5	89
V	18.5	15.7	14.7	32.2	26.8	27.8	6.7	19.6	1.2	258
VI	15.0	14.0	12.5	27.5	21.6	16.4	5.2	15.3	2.6	130
VII	23.5	22.1	21.4	32.1	23.0	14.0	4.2	21.8	3.2	57
Mean	19.9 ± 7.5	16.9 ± 5.0	15.5 ± 5.8	27.0 ± 7.5	21.5 ± 7.1	15.8 ± 7.3	5.6 ± 3.3	19.2 ± 16.0	4.8 ± 6.1	195.6 ± 249.6

**Table 3:** Dose to organs at risk for VMAT plans for all seven patients

Patient no	VMAT plan									
	Cord max (Gy)	Cord 0.3 cc (Gy)	Cord 1.2 cc (Gy)	Esophagus max (Gy)	Esophagus 5 cc (Gy)	Heart max (Gy)	Heart 15 cc (Gy)	Lung V5 (cc)	Lung V10 (cc)	Lung D1000 cc (cGy)
I	11.7	10.3	6.7	12.9	7.4	10.8	6.7	10.7	2.8	71
II	21.0	19.4	17.0	25.4	20.0	2.1	5.2	6.9	2.8	19
III	24.3	23.1	22.3	32.8	29.3	21.4	10.9	52.8	18.8	729
IV	14.8	12.7	11.8	19.9	15.1	12.5	2.6	7.1	1.8	50
V	16.8	14.1	13.4	26.4	21.8	20.9	6.7	21.8	1.1	146
VI	14.4	12.9	12.6	18.5	14.4	11.8	4.4	12.2	1.3	80
VII	21.6	17.8	15.3	21.9	15.6	12.2	3.9	18.2	2.7	56
Mean	17.8 ± 4.6	15.7 ± 4.5	14.2 ± 4.8	22.5 ± 36.4	17.7 ± 6.9	13.1 ± 6.6	5.1 ± 3.4	18.5 ± 16.1	4.5 ± 6.4	164.4 ± 252.0

Table 4 shows detailed information on total number of MUs, BOT, and QA passing rate values for hybrid and VMAT plans. Data are presented as the mean and standard deviations for all seven patients. For VMAT plans, the total number of MUs was 2598 ± 345 on average compared to 3542 ± 495 for hybrid plans (*P* = 0.003). This reduction in MUs translated into lower BOTs from an average of 7.1 ± 1.0 min to 4.7 ± 0.6 min (*P* < 0.001).

**Table 4:** Detailed information on average total number of MUs, beam‐on time, and IMAT QA pass rate values for Hybrid and VMAT plans

Patient no	Hybrid plan		VMAT plan	
	Monitor units	Beam‐on time (min)	Monitor units	Beam‐on time (min)
I	4152	8.30	3216	5.63
II	3123	6.25	2651	4.84
III	2739	5.48	2437	4.27
IV	3949	7.90	2145	3.76
V	3353	6.71	2635	4.81
VI	3756	7.51	2297	4.19
VII	3720	7.44	2808	5.12
Mean	3542 ± 495	7.08 ± 0.99	2598 ± 354	4.66 ± 0.63

## 4 DISCUSSION

In this study, we have presented a dosimetric comparison of two methods of delivering SBRT for thoracic vertebral metastases: (a) noncoplanar hybrid arcs consisting of 1‐2 partial arcs and 7‐9 static IMRT beams and (b) VMAT with three full coplanar arcs. We found that VMAT plans provided improved dose distributions and improved normal tissue sparing with statistically significant improvements in heart and esophagus doses. VMAT plans were twice as homogeneous as hybrid plans as indicated by the homogeneity index (0.14 vs. 0.27, *P* = 0.01).

Homogeneous dose delivery is especially important in SBRT treatment of the thoracic spine due to the proximity of the spinal cord to the vertebral bodies, unlike in lung SBRT where heterogeneous dose is desired. Less homogeneous plans will have more variation between hot and cold spots. Due to setup uncertainties, such heterogeneous plans with multiple hot spots in the vicinity of the spinal cord are undesirable because intrafraction (patient motion) and interfraction (daily setup errors) variation may result in an adjacent hot spot on the spinal cord. Because of this uncertainty and threat of overdosing the spinal cord, most physicians will opt for fractionated treatment instead of single fraction treatment. We have previously published on the use of single fraction stereotactic body radiosurgery (SBRS) to the spine using VMAT and demonstrated that such treatment was feasible following RTOG 0631 dosimetric criteria with compliant spinal cord doses and highly conformal and homogeneous dose distributions.^32^, ^33^ However, there remain a number of concerns regarding single fraction treatment in regard to normal tissue dosing and treatment logistics.

Esophageal dose has been demonstrated to be particularly problematic for single fraction treatments.^34^, ^35^ Cox et al. studied SBRS treatment in 182 patients with tumors abutting the esophagus and found a 6.8% rate of acute or late grade 3 + esophageal toxicity. The median dose in this study was 24 Gy, and the median follow‐up time was 12 months. A dose–response model was generated, which revealed sharply increasing rates of toxicity when the dose to the hottest 2.5 cc of the esophagus (D2.5 cc) was greater than 14 Gy. Under 14 Gy, the risk of grade 3 + toxicity was <5%, but this risk increased to 15% at a D2.5 cc of 20 Gy.^34^ The esophageal dose constraints on the RTOG 0631 protocol were D0.3 cc < 18 Gy, and D5 cc < 11.9 Gy.^33^ In our single fraction study, the maximum dose to the esophagus was 11.2 Gy on average (range 7.1–14.9 Gy), which was well below the protocol requirement.^32^ In the current study with 5 fraction SBRT, the average maximum esophagus dose with VMAT plans was 22.5 Gy, which equates to a biological effective dose (BED) of 56.3 Gy using an alpha/beta ratio of 3 for late toxicity. The average D5 cc was 17.6 Gy, which has a BED of 38.3 Gy. These were similar to the maximum and D5 cc doses on the single fraction study, which were 53.0 and 36.7 Gy BED, respectively. However, the BED of the maximum and D5 cc doses for the hybrid plans on the current study were much higher at 75.6 and 52.3 Gy, respectively. Our results suggest that fractionated VMAT plans offer significantly reduced esophageal toxicity compared to hybrid plans. Effective doses to the esophagus are similar compared to single fraction plans but come with the benefit of a wider therapeutic ratio conferred by delivering the dose over multiple fractions.

Treatment time is another major clinical consideration in the delivery of spinal SBRT. Beam‐on times for single fraction radiosurgery to spinal lesions have been reported for various modalities in a recent study by Nalichowski et al., ranging from as low as 4.4 min with flattening filter free (FFF) RapidArc to 58.1 min with CyberKnife.^36^ The authors reported that CyberKnife was the only modality that used noncoplanar beams and produced the lowest spinal cord doses and best conformality; however, these benefits came at the cost of much longer treatment times. In our study, we found that VMAT allowed for faster delivery of radiation compared to noncoplanar hybrid plans while actually improving conformality and normal tissue sparing. The average beam‐on time was 4.7 min for VMAT compared 7.1 min for hybrid plans (not accounting for couch kicks). In actuality, effective treatment times for hybrid plans using noncoplanar arcs are much longer due to required repositioning of the table (couch kicks) by the therapist during treatment. For the patient population with spinal metastases undergoing palliative radiation, prolonged treatment times can cause significantly more pain and discomfort, which can also result in additional patient movement and aborted treatments. Our results demonstrate that VMAT allows for faster treatments through the use of coplanar arcs without sacrificing target coverage and OAR sparing.

Wu et al. have previously reported a series of 10 patients treated with SBRT to the spine using either static IMRT or VMAT plans (with one or two arcs). When only one arc was used, VMAT plans were significantly worse in terms of spinal cord dosing, but there was no difference with two arcs. The mean treatment times were improved with VMAT plans (6.38 min beam‐on time for 2 arc plans). In our study, we used three coplanar arcs for VMAT plans, and found similarly improved treatment efficiency without any significant difference in spinal cord sparing.^37^

## 5 CONCLUSION

In this paper, we have presented the results of our study investigating the feasibility of using VMAT for SBRT spine treatments instead of noncoplanar hybrid arcs. VMAT plans resulted in improved dose distributions and normal tissue sparing compared to NCHA plans. Treatment times were significantly shorter with VMAT plans, which is advantageous in both clinical efficiency as well as minimizing patient discomfort and intrafraction movement error. VMAT planning using SBRT is an attractive option for the treatment of metastases to thoracic vertebrae, and further investigations using alternative fractionation schedules are warranted.

## CONFLICTS OF INTEREST

The authors do not have any conflicts of interest to declare.
